# Evaluation of foundational mathematics support for health sciences students

**DOI:** 10.1186/s12909-026-10151-8

**Published:** 2026-08-18

**Authors:** Yusuke Ono, Yumiko Abe, Yasumasa Totsuka, Hirokazu Murakami

**Affiliations:** 1 Department of Medical Technology and Clinical Engineering, Gunma University of Health and Welfare, 191-1, Kawamagari-machi, Maebashi, Gunma 3710823 Japan; 2 The Support Center for Higher Education, Gunma University of Health and Welfare, 191-1, Kawamagari-machi, Maebashi, Gunma 3710823 Japan

**Keywords:** Remedial education, Mathematics education, Health sciences students, Learning outcomes, Medical technology and clinical engineering

## Abstract

**Background:**

University entrants have increasingly diverse academic backgrounds, creating a need for foundational mathematics support in health sciences programs. In medical technology and clinical engineering education, mathematical competence underpins subjects such as statistics, physics, and engineering, as well as clinically relevant quantitative tasks. This study evaluated a mathematics remedial education program for first-year undergraduate students in the Department of Medical Technology and Clinical Engineering and examined unit-specific learning gains.

**Methods:**

Participants were first-year students in two academic years (2024: *n = 28*; 2025: *n = 30*). Pre- and post-tests covering fundamental mathematical topics were administered before and after the program. Differences in percentage scores were analyzed using paired t-tests, and effect sizes were calculated using Cohen’s *d*.

**Results:**

Overall scores improved significantly in both academic years. In the pooled sample, the mean score increased from *28.00 ± 11.11* to *61.36 ± 18.89* (mean difference: 33.36 points, 95% CI: 29.06–37.66; *p < 0.001*; Cohen’s *d* = 2.04). Statistically significant improvements were observed in 10 of the 11 mathematical units, although the magnitude of improvement varied by unit.

**Conclusions:**

Early foundational mathematics support was associated with improved test performance and may contribute to students’ academic readiness for quantitatively demanding health science coursework. Unit-specific evaluation may help identify areas requiring additional instructional support and inform the design of transition-stage learning support in health science education.

## Background

In recent years, the academic backgrounds of students entering higher education have become increasingly diverse, and some students begin university without the foundational mathematical knowledge required for successful transition to university-level study [20]. In response to this situation, many educational institutions have implemented remedial or developmental education programs [4, 6, 8]. In mathematics education in particular, recent course redesigns and targeted support initiatives have continued to address differences in incoming students’ mathematical preparation and to improve progression into college-level mathematics [1, 5]. However, the effectiveness of remedial education may vary according to program design, instructional approach, and student characteristics [2, 3].

Basic mathematical skills are important not only in science and engineering fields but also in health sciences, where quantitative reasoning is required for tasks such as calculating drug dosages, determining concentrations, and interpreting statistical data. In particular, insufficient numerical ability may affect students’ performance in clinically relevant calculations and their understanding of specialized subjects [15, 17, 29]. Recent research has also highlighted persistent difficulties in clinically related numeracy and the potential value of structured, contextualized mathematical support in undergraduate health science education [15, 19]. These findings highlight the need to support foundational mathematical learning during the early stages of health science education.

The pre-test/post-test design, which compares learning outcomes before and after an educational intervention, is widely used as a method for evaluating instructional effectiveness [10, 11]. This method allows for quantitative assessment of changes associated with educational programs, and gain scores are often used as an indicator of pre–post change [11]. In addition to statistical significance, reporting effect sizes is important for interpreting the practical magnitude of observed differences [9, 12]. Recent reporting guidance for quantitative educational intervention research also recommends presenting effect estimates together with measures of precision, such as 95% confidence intervals [27]. At the same time, changes in average scores alone may not provide a sufficient understanding of educational effectiveness. In particular, the effects of educational interventions may vary depending on learners’ prior academic proficiency, making stratified analysis important [7, 24]. Furthermore, in mathematics education, both conceptual understanding and procedural skills influence learning outcomes, suggesting the importance of analyzing performance at the level of individual content units [21, 26].

Several analytical approaches have been used to examine educational outcomes from multiple perspectives. When multiple comparisons are conducted, correction methods such as the Holm method are used to control Type I errors [13]. In addition, principal component analysis (PCA) has been used to explore relationships among multiple assessment items and to examine the structure of educational evaluation data [14]. Beyond quantitative evaluation, the design of foundational skills instruction should also consider its connection to disciplinary contexts. Contextualized basic skills instruction has been proposed as a way to connect foundational skills such as reading, writing, and mathematics with disciplinary or occupational contexts, thereby supporting transfer of learning and student motivation [18]. More recent research has also reported positive associations between exposure to contextualized mathematics instruction and a range of academic outcomes in postsecondary education [28]. This perspective is also relevant to the development of remedial mathematics programs in health science education.

At the Department of Medical Technology and Clinical Engineering of our university, a mathematics placement test is administered upon admission to assess students’ foundational mathematical skills. This test covers fundamental mathematical topics required for subsequent specialized medical courses, including algebraic calculations, equations, logarithmic calculations, trigonometric functions, and concentration calculations. Based on the results of this placement test, remedial education is provided to students in the lower range of the score distribution who are considered to require additional support.

Therefore, this study aims to evaluate a mathematics remedial education program for first-year students in the Department of Medical Technology and Clinical Engineering. Specifically, we analyze changes in test scores before and after the program and differences in learning gains across mathematical units and across initial proficiency groups.

## Methods

### Participants

The participants in this study were first-year students who enrolled in the Department of Medical Technology and Clinical Engineering at our university in the 2024 and 2025 academic years and participated in the mathematics remedial education program. A total of 58 students were included, comprising 28 students in 2024 and 30 students in 2025. They were enrolled in either the Clinical Engineering program or the Medical Technology program.

Eligibility for the remedial program was determined using the mathematics placement test administered at the time of enrollment, which also served as the pre-test in this study. The maximum program capacity was set at 30 students for practical instructional reasons. In 2024, participation was strongly recommended for students who scored below 40% on the placement test. In addition, five students who scored 40% or higher and wished to strengthen their foundational mathematical skills participated voluntarily.

In 2025, the number of students scoring below 40% was expected to exceed the program capacity. Therefore, participation was strongly recommended for students who scored below 30%. Students who scored 30% or higher but below 40% were permitted to participate based on their willingness to do so, and two students joined the program through this route.

All participants completed both the pre-test and post-test and were included in the paired analysis. Regardless of the selection route, all participants received the same instruction and completed the same assessments under the same conditions.

All participants provided informed consent for the use of their anonymized data in this study. To protect personal information, all student data were anonymized before analysis, and the analytical dataset contained no directly identifiable information.

This study was approved by the institutional ethics review board (Approval Nos. RS23-22 and RS24-26).

### Remedial program

The remedial education program was implemented shortly after university enrollment to strengthen the foundational mathematical knowledge and skills required for subsequent coursework in the Medical Technology and Clinical Engineering programs. In the 2024 academic year, the program was conducted from April 17 to May 29, once a week on Wednesdays, with each session lasting 90 minutes. In the 2025 academic year, it was conducted from April 16 to June 4 using the same weekly schedule. The program consisted of seven sessions in each academic year.

The course content was selected in consideration of its relevance to subsequent disciplinary learning. For example, concentration calculations provide a mathematical foundation for chemistry, solution preparation associated with life-support equipment in the Clinical Engineering program, and reagent preparation in the Medical Technology program. Trigonometric ratios likewise provide a foundation for determining distances and angles in physics- and engineering-related subjects. Representative assessment questions illustrating these applications are provided in Appendix A.

The program primarily aimed to reinforce foundational computational proficiency. However, selected topics and exercises also required students to interpret quantitative relationships, apply mathematical knowledge to contextualized problems, and use basic mathematical reasoning and problem-solving skills.

Each 90-minute session consisted of approximately equal periods of instruction and practice. During the instructional component, the instructor explained fundamental concepts and demonstrated worked examples. During the practice component, students solved exercises individually and were encouraged to discuss their approaches with peers seated nearby. When questions or common difficulties arose during the exercises, the instructor provided additional explanations to the whole class. Thus, although the program did not employ a formal problem-based or differentiated learning model, it incorporated guided practice, informal peer discussion, and responsive whole-class feedback.

The same external instructor taught the program in both academic years. The instructional structure, teaching materials, session duration, and core course content were maintained across the two years to ensure consistency of the intervention.

Attendance was recorded for all sessions. In 2024, 21 of the 28 participants attended all seven sessions, six missed one session, and one missed two sessions. In 2025, 22 of the 30 participants attended all seven sessions, three missed one session, one missed two sessions, and four missed three sessions. Students with one or more absences were retained in the analysis because the study evaluated the outcomes of all participants enrolled in the program who completed both the pre-test and post-test.

### Test design

To evaluate pre–post changes associated with the remedial education program, tests with identical structures were administered before and after the program. The pre-test corresponded to the mathematics placement test administered at the time of enrollment, and the post-test was administered after completion of the program. The two tests covered the same mathematical domains and contained the same numbers and types of items, but used different questions. In the 2024 academic year, the pre-test and post-test were administered on April 5 and June 14, respectively. In the 2025 academic year, they were administered on April 4 and June 11, respectively. The test consisted of 11 questions, each containing multiple sub-questions.

The test content was as follows:

- Question 1: Algebraic calculations (4 items)
- Question 2: Equations (2 items)
- Question 3: Logarithmic calculations (2 items)
- Question 4: Exponential and logarithmic expressions (2 items)
- Question 5: Rationalization and complex number operations (1 item each)
- Question 6: Systems of equations (2 items)
- Question 7: Trigonometric functions (2 items)
- Question 8: Linear functions (3 items)
- Question 9: Logical reasoning problem (1 item)
- Question 10: Concentration calculations (3 items)
- Question 11: Trigonometric ratios (1 item)

The assessment primarily focused on foundational computational and procedural skills, including algebraic, logarithmic, and trigonometric calculations. However, selected items also assessed students’ ability to interpret quantitative relationships, apply mathematical knowledge to contextualized problems, and use basic logical reasoning and problem-solving skills. For example, Question 9 assessed logical reasoning, while Questions 10 and 11 required the application of mathematical knowledge to chemistry- and physics-related situations, respectively. Representative assessment questions are provided in Appendix A. Each sub-question was scored as 1 point for a correct answer and 0 points for an incorrect answer. In cases where partial credit was awarded, intermediate scores such as 0.5 points were assigned. Each student’s percentage score was calculated as the ratio of the obtained score to the total possible score (*%*).

1 $$\begin{aligned} \textrm{ScoreRate}_i = \frac{S_i}{S_{\max }} \times 100 \end{aligned}$$

where $S_i$ is the total score obtained by student *i*, and $S_{\max }$ is the maximum possible score.

The pre-test and post-test shared identical structures, ensuring comparability of results and enabling analysis of pre–post changes.

### Stratification method

To examine whether learning gains differed according to initial mathematical proficiency, participants were stratified based on their pre-test percentage scores. Stratification was performed using percentile thresholds. Participants were classified into three groups: Low, Middle, and High. Specifically, within each academic year, the 33rd and 67th percentiles of the pre-test score distribution were used as cutoff values.

Using this classification, pre–post changes and gain scores were compared across the three initial-proficiency groups. Participants who joined the program based on their willingness to participate were not treated as a separate analytical group. Instead, they were classified into the Low, Middle, or High group using the same pre-test percentile thresholds applied to all other participants.

### Statistical analysis

The pre-test/post-test design is widely used to assess changes in learning outcomes resulting from educational interventions [10]. To evaluate pre–post changes associated with the remedial education program, paired *t*-tests were conducted on differences in percentage scores between the pre-test and post-test. Statistical analyses were performed separately for each academic year and for the pooled sample, with the significance level set at 5%. Two-sided 95% confidence intervals were calculated for the mean pre–post differences.

Furthermore, to assess the magnitude of the pre–post difference, Cohen’s *d* was calculated as a measure of effect size [9]. For paired comparisons, Cohen’s d was calculated as the mean of the difference scores divided by the standard deviation of the difference scores.

2 $$\begin{aligned} d = \frac{\bar{x}_{post}-\bar{x}_{pre}}{s_d} \end{aligned}$$

where $s_d$ is the standard deviation of the difference between pre-test and post-test scores.

To assess pre–post changes associated with the remedial education program, paired *t*-tests were conducted for the pre-test and post-test correct response rates of each question. The Holm method was applied to adjust for multiple comparisons across the 11 questions. Both raw and Holm-adjusted *p*-values were calculated, and statistical significance in the unit-level analysis was determined based on the Holm-adjusted *p*-values. In the stratified analysis, the mean and standard deviation of percentage scores were calculated for each group, and changes before and after the intervention were evaluated. To compare the magnitude of improvement among the initial proficiency groups, one-way analysis of variance (ANOVA) was conducted using gain score as the dependent variable and initial proficiency group as the factor.

The assumptions underlying the parametric analyses were examined before statistical testing. The normality of paired difference scores and ANOVA residuals was assessed using the Shapiro–Wilk test, and homogeneity of variance across the initial proficiency groups was assessed using Levene’s test. The paired difference scores did not show significant deviations from normality in 2024 (*W = 0.956*, *p = 0.284*), 2025 (*W = 0.945*, *p = 0.125*), or the pooled sample (*W = 0.981*, *p = 0.510*). For the pooled gain-score ANOVA, the residuals did not show a significant deviation from normality (*W = 0.983*, *p = 0.606*), and Levene’s test did not indicate a significant violation of homogeneity of variance (*F = 1.77*, *p = 0.180*).

As a supplementary exploratory analysis, principal component analysis (PCA) was conducted using standardized unit-level pre-test correct response rates to examine the structure of baseline mathematical performance. The unit-level variables were standardized to have a mean of zero and a standard deviation of one before PCA. PCA was used to explore whether the initial proficiency groups reflected overall performance level or differences in performance patterns across units. The results were interpreted descriptively using the score plot and scree plot.

All statistical analyses were conducted using Python with the NumPy, SciPy, pandas, statsmodels, and scikit-learn libraries.

## Results

### Overall improvement

To evaluate pre–post changes in test performance, pre-test and post-test scores were compared. Table 1 summarizes the descriptive statistics and results of the paired t-tests. For the entire sample (*n = 58*), the mean post-test score (*61.36 ± 18.89*) was substantially higher than the mean pre-test score (*28.00 ± 11.11*), indicating a statistically significant improvement (*t(57) = 15.54*, *p < 0.001*). The mean gain score was 33.36 points, and the effect size for the pre–post difference was large (*d = 2.04*).

**Table 1:** Pre–post comparison by academic year (AY)

AY	*n*	Pre ($\boldsymbol{\mu }\ \boldsymbol{\pm }$SD)	Post ($\boldsymbol{\mu }\ \boldsymbol{\pm }$SD)	Mean Gain (95% CI)	*t*	*p*	Cohen’s *d*
2024	28	*31.64 ± 12.59*	*62.82 ± 21.55*	31.18 (24.23–38.13)	9.21	*p<0.001*	1.74
2025	30	*24.60 ± 8.38*	*60.00 ± 16.26*	35.40 (29.89–40.91)	13.15	*p<0.001*	2.40
All	58	*28.00 ± 11.11*	*61.36 ± 18.89*	33.36 (29.06–37.66)	15.54	*p<0.001*	2.04

A similar trend was observed across both academic years. In 2024 (*n = 28*), the mean score increased from *31.64 ± 12.59* to *62.82 ± 21.55*, with a mean gain of 31.18 points (*t(27) = 9.21*, *p < 0.001*, *d = 1.74*). In 2025 (*n = 30*), the mean score increased from *24.60 ± 8.38* to *60.00 ± 16.26*, corresponding to a mean gain of 35.40 points (*t(29) = 13.15*, *p < 0.001*, *d = 2.40*).

**Fig. 1:**
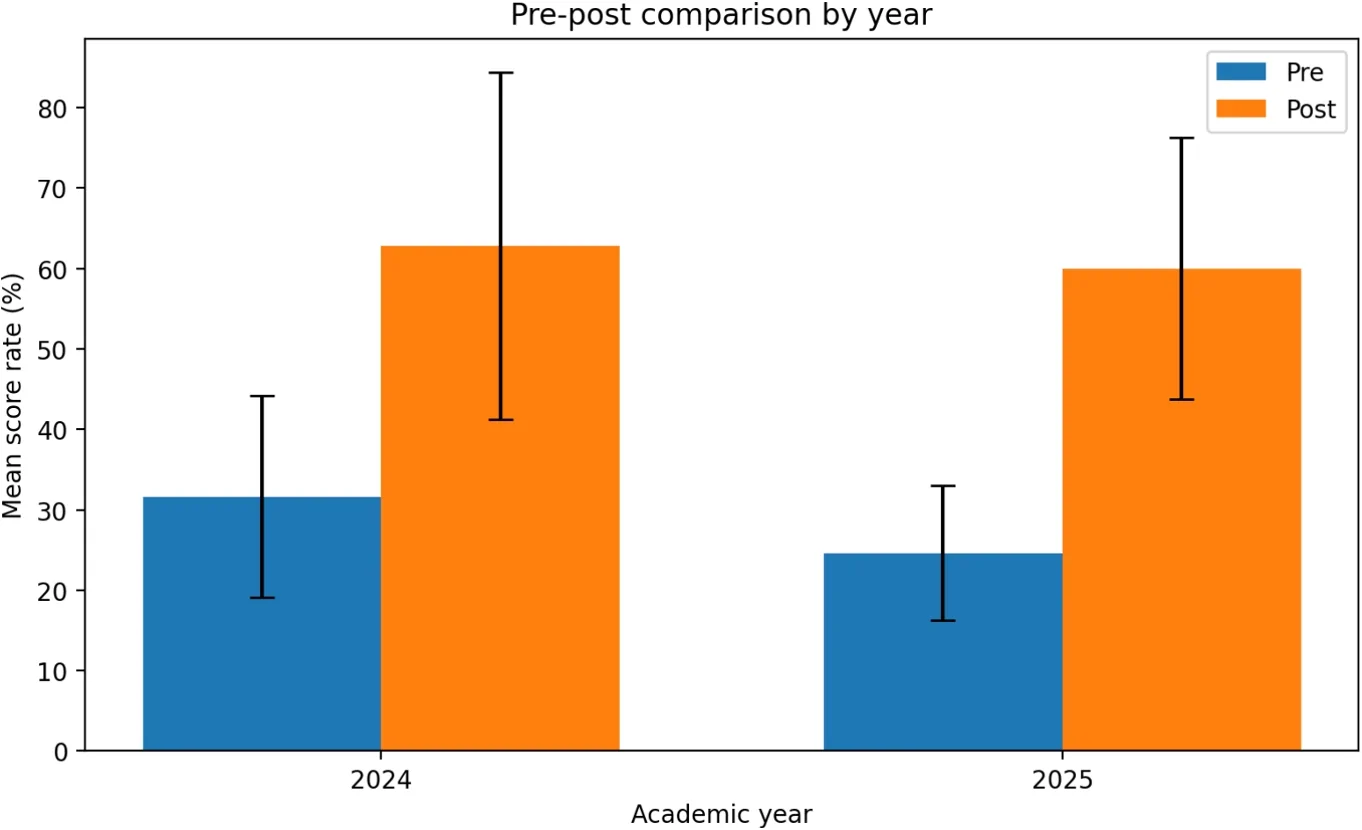
Pre–post comparison of mean score rates by academic year. Error bars indicate standard deviations

Figure 1 visually shows the increase in mean score rates from pre-test to post-test in both academic years. Together with the large effect sizes, these results indicate that the remedial program was associated with substantial increases in foundational mathematics test performance.

### Stratified analysis

Participants were classified into three groups—Low, Middle, and High—based on their pre-test scores, and the magnitude of improvement was compared across groups. In all groups, the difference between pre-test and post-test scores was statistically significant: Low group: *t(19) = 7.87*, *p < 0.001*, *d = 1.76*; Middle group: *t(18) = 7.91*, *p < 0.001*, *d = 1.82*; High group: *t(18) = 12.15*, *p < 0.001*, *d = 2.79*. The mean gain scores were 32.70 points for the Low group, 32.58 points for the Middle group, and 34.84 points for the High group, indicating large effect sizes across all groups.

**Fig. 2:**
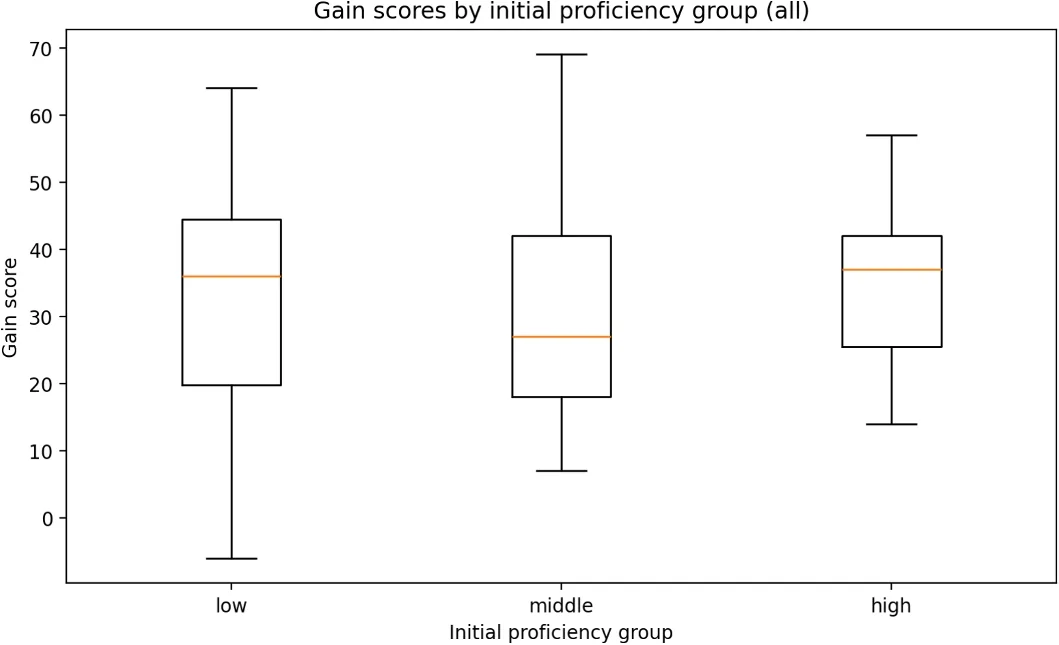
Distribution of gain scores by initial proficiency group for all participants

A one-way analysis of variance (ANOVA) revealed no statistically significant difference in gain scores among the three groups (*F(2,55)=0.11*, *p=0.894*, partial $\eta ^2=0.004$). Figure 2 shows the distribution of gain scores across the three groups. The medians are comparable across groups, and the interquartile ranges largely overlap, indicating broadly comparable gain-score distributions across the three groups. Although variability within each group was observed, the median gain scores were relatively similar across groups, supporting the finding that no statistically significant differences were present.

These results indicate that pre–post improvements were observed across all three initial proficiency groups, with no statistically significant differences in gain scores among the groups. Detailed results for each academic year are provided in Appendix B (Figs. 5 and 6).

As a supplementary analysis, principal component analysis (PCA) was conducted using unit-level pre-test correct response rates. PCA is widely used to explore the latent structure of multivariate data [14]. The results indicated that the first principal component (PC1) primarily reflected overall performance level, and the Low, Middle, and High groups tended to be distributed along this axis. However, some overlap in the distributions across groups was observed, suggesting that learners’ understanding structures may not be fully represented by a simple one-dimensional scale. The PCA score plot and scree plot are provided in Appendix B (Figs. 7 and 8).

### Unit-level analysis

To examine changes in performance at the unit level, we analyzed differences in correct response rates for each question (Q1–Q11). To control for Type I error due to multiple comparisons, the Holm correction was applied [13]. Statistically significant improvements based on the Holm-adjusted *p*-values were observed in 10 of the 11 units (adjusted *p < 0.05*). Although Q1 showed an increase in correct response rate, the improvement did not reach statistical significance after Holm adjustment (raw *p = 0.060*, adjusted *p = 0.060*, *d = 0.25*).

**Fig. 3:**
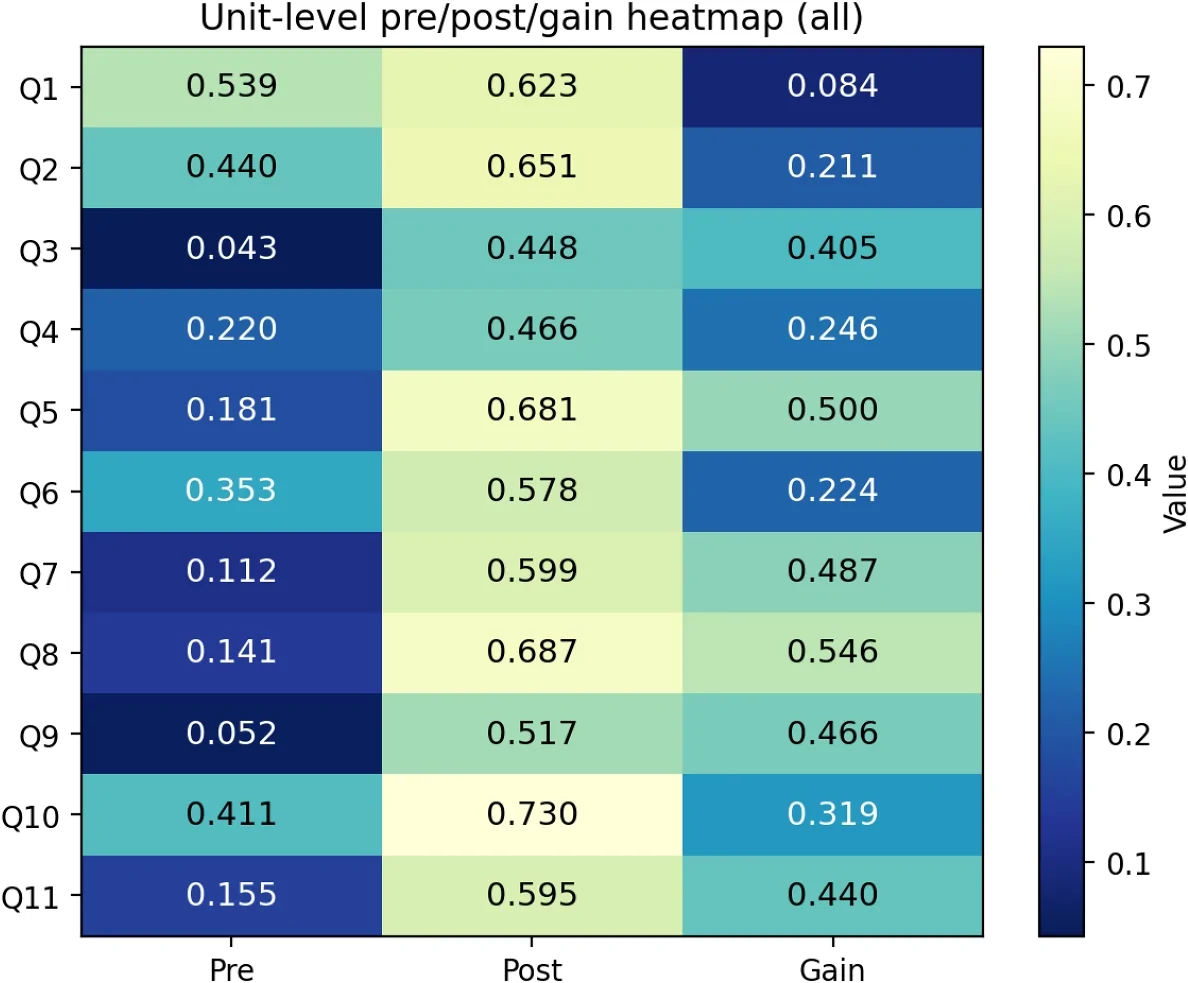
Unit-level comparison of pre-test, post-test, and gain scores. Values represent mean correct response rates for each question (Q1–Q11)

**Fig. 5:**
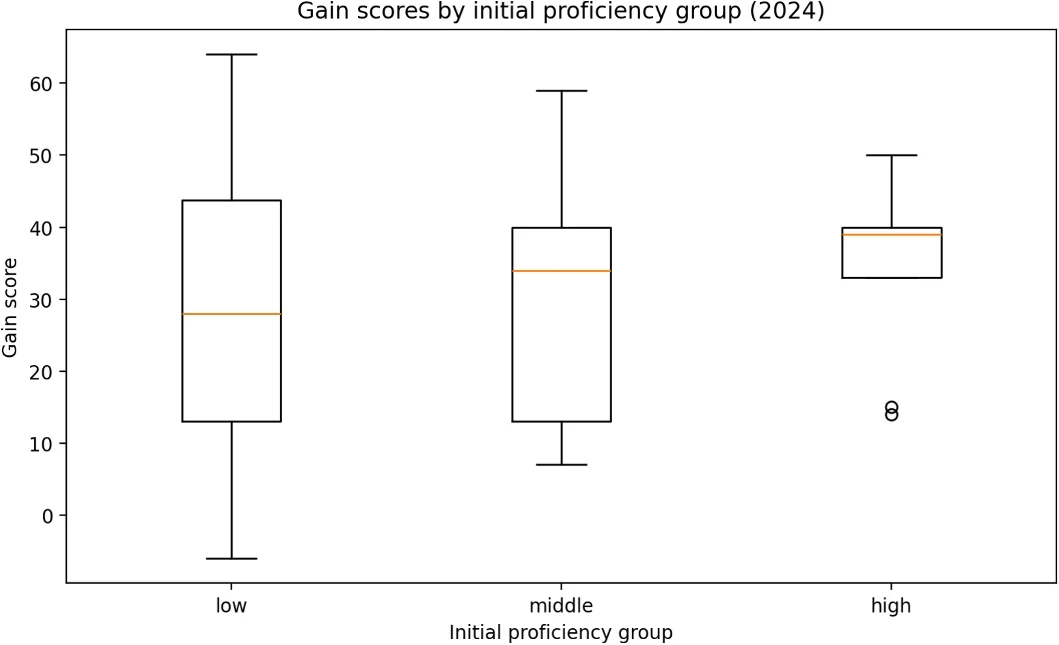
Distribution of gain scores by initial proficiency group in the 2024 academic year

**Fig. 6:**
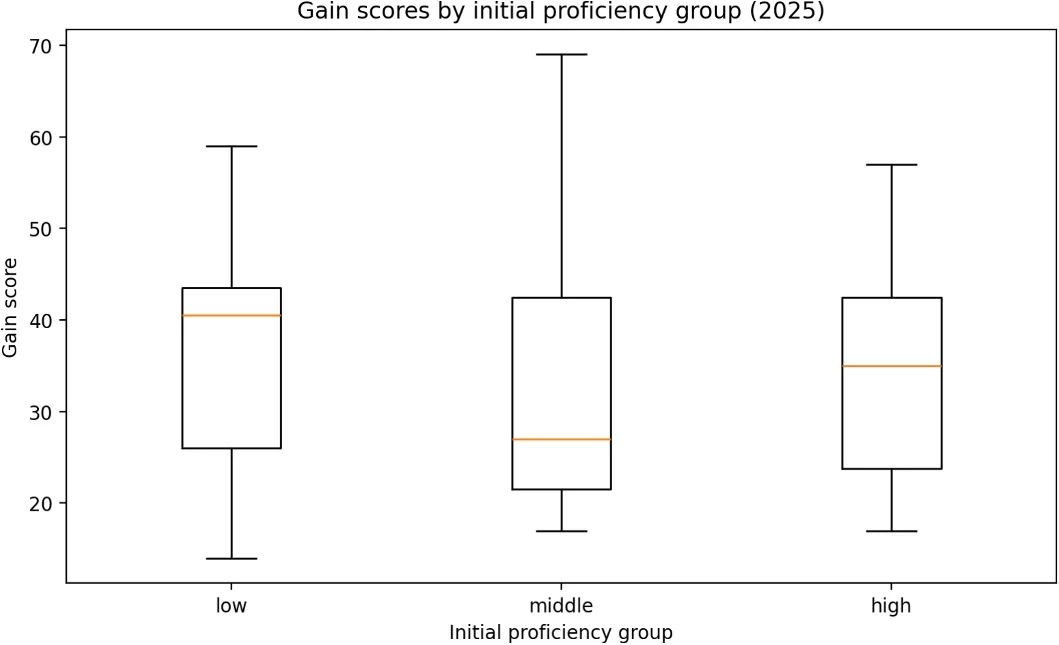
Distribution of gain scores by initial proficiency group in the 2025 academic year

**Fig. 7:**
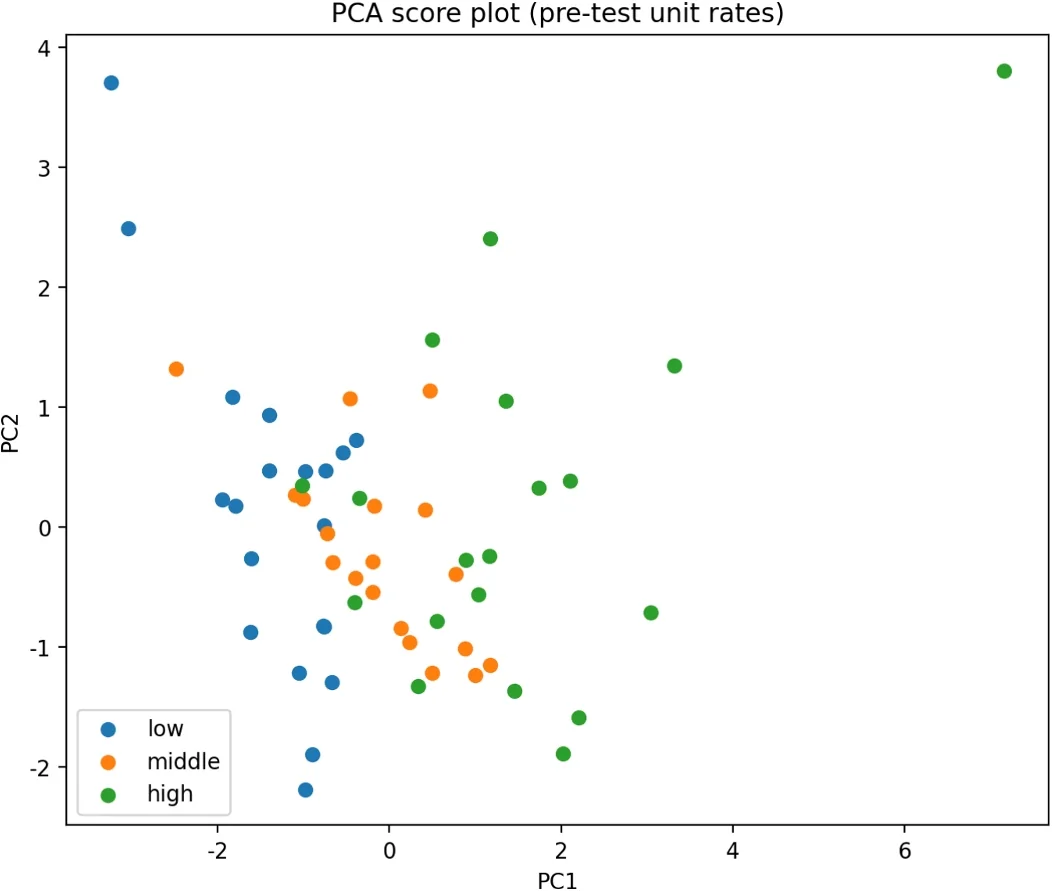
PCA score plot based on pre-test unit-level performance

**Fig. 8:**
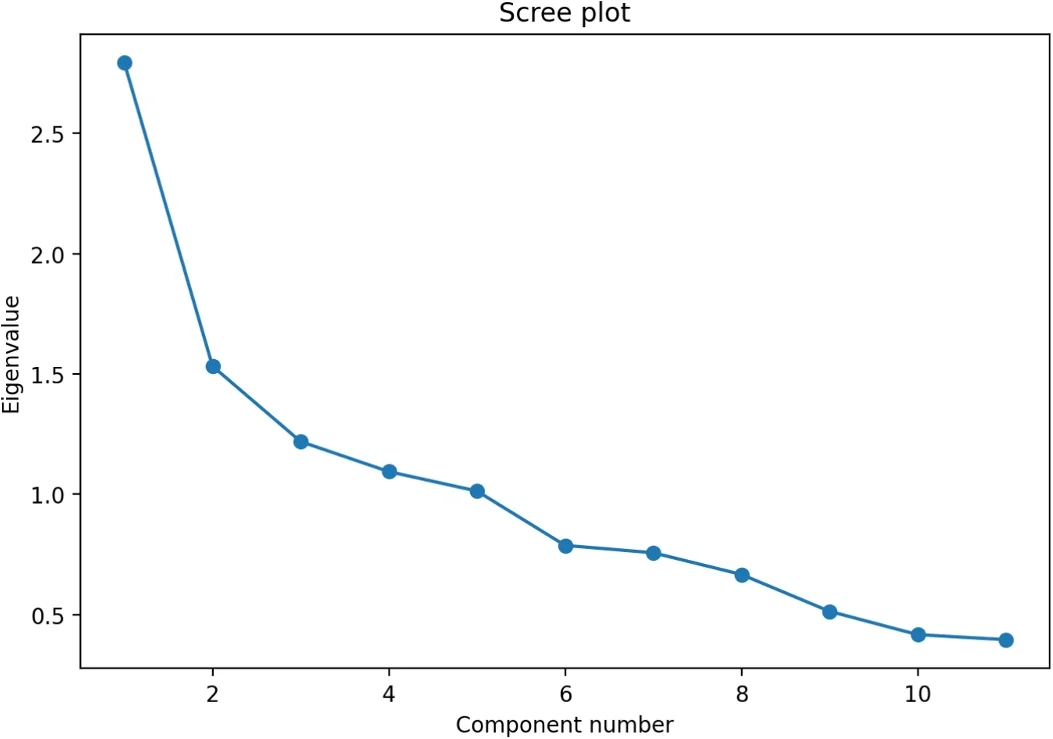
Scree plot showing eigenvalues of principal components

Figure 3 presents the pre-test, post-test, and gain scores for each unit. Improvements in correct response rates were observed across most units, indicating that the observed gains extended across multiple content areas. In particular, relatively large improvements were observed in Q5, Q7, and Q8, which correspond to rationalization/complex numbers, trigonometric functions, and linear functions. These units showed some of the largest observed increases in correct response rates and primarily assessed foundational computational skills. Furthermore, larger gains tended to occur in units with lower pre-test performance.

Despite substantial gains, post-test performance remained relatively low for Q3 and Q4, with correct response rates of 44.8% and 46.6%, respectively. These items required students to apply multiple properties of logarithms and exponential expressions in combination.

**Fig. 9:**
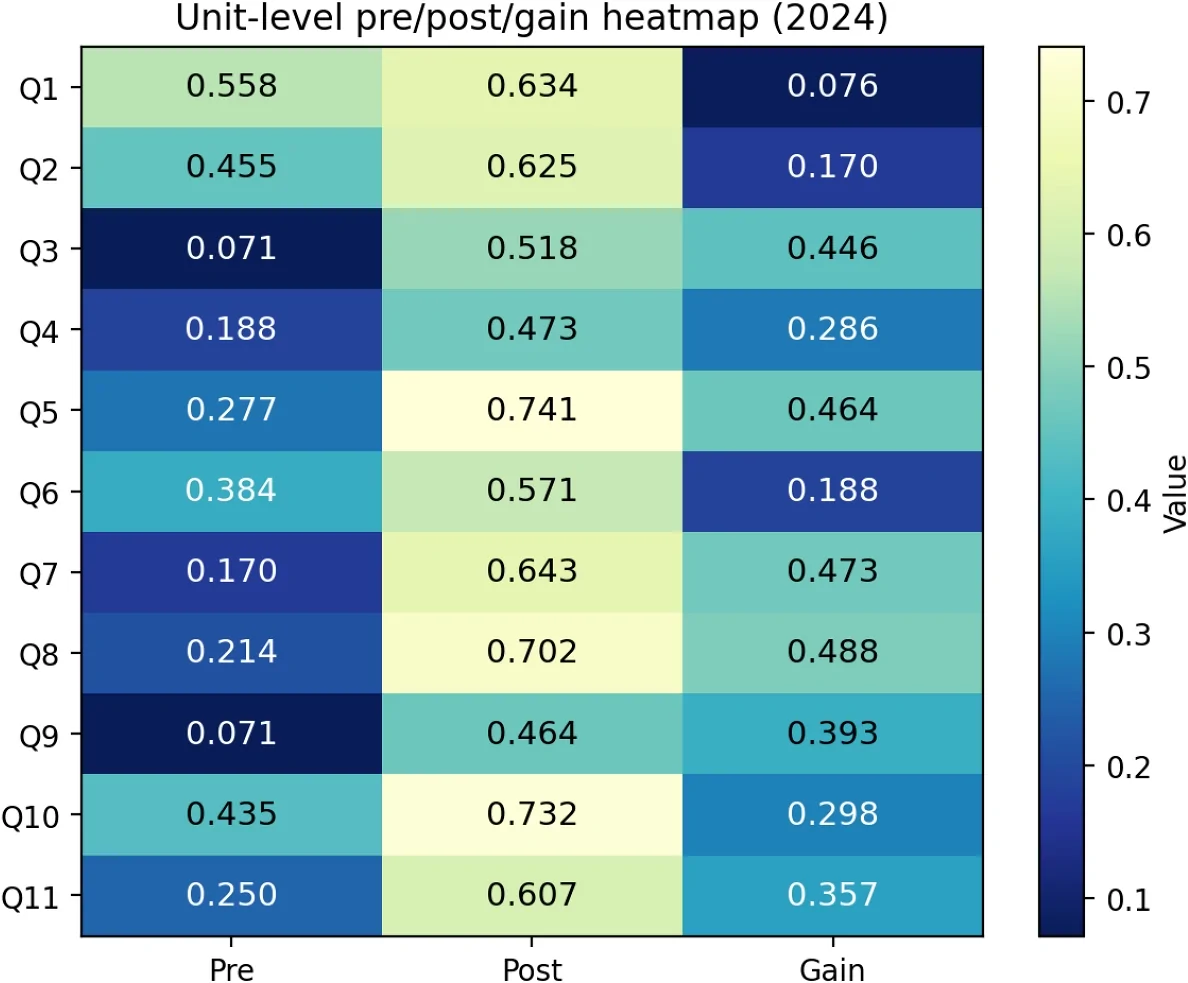
Unit-level pre/post/gain heatmap for the 2024 academic year

**Fig. 10:**
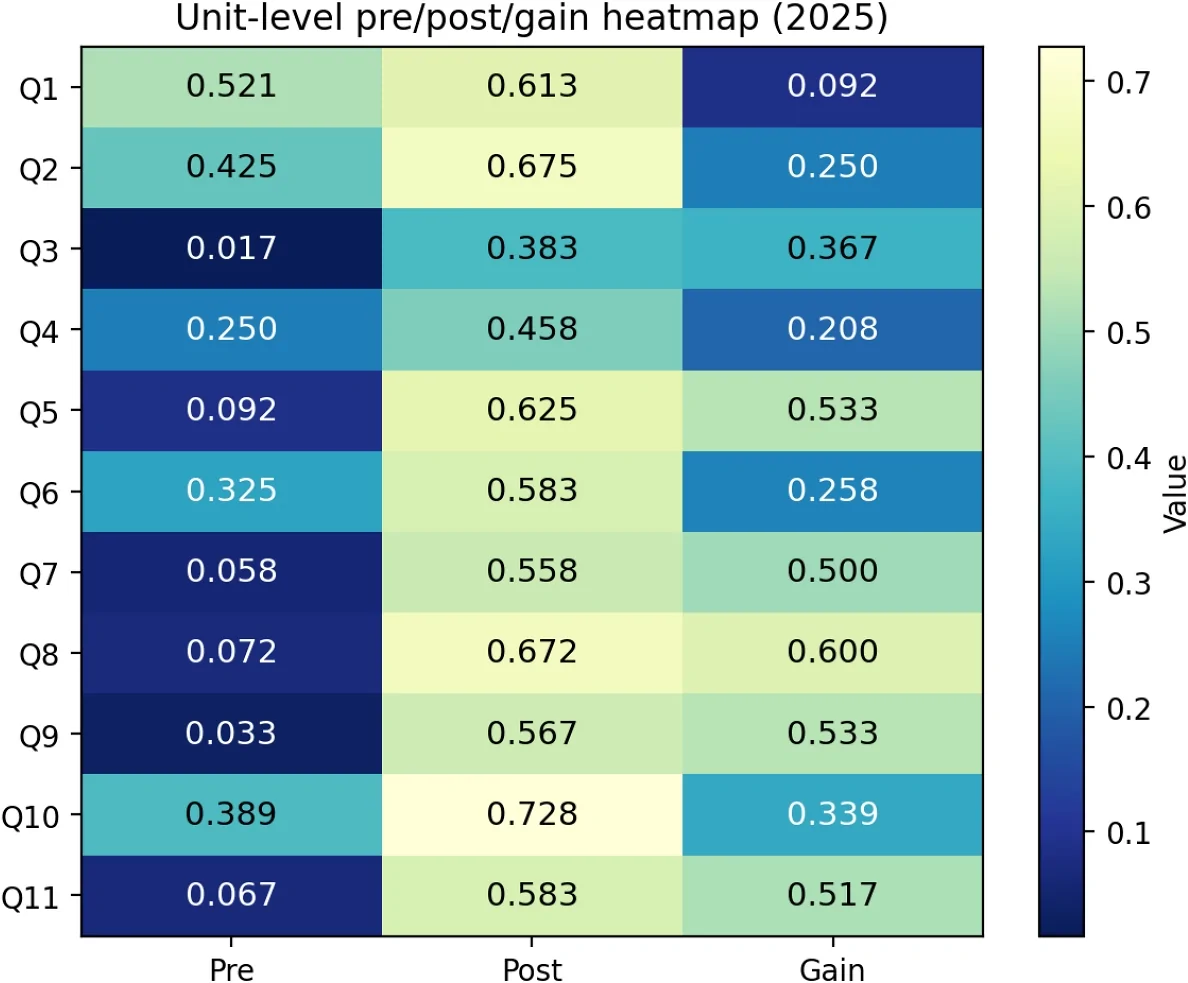
Unit-level pre/post/gain heatmap for the 2025 academic year

Overall, these findings indicate that the remedial program was associated with improvements across a broad range of mathematical units rather than affecting only specific units. Complete unit-level results for the pooled sample, including raw and Holm-adjusted *p*-values, are provided in Appendix B (Table 2). Detailed unit-level results for each academic year are also provided in Appendix B (Figs. 9 and 10).

## Discussion

### Overall evaluation of the remedial program

In this study, we examined pre–post changes associated with remedial mathematics instruction in the Department of Medical Technology and Clinical Engineering using pre-test and post-test results. The results showed a statistically significant overall increase in scores, accompanied by a large standardized pre–post difference. These findings are broadly consistent with previous studies reporting improved learning outcomes following remedial or developmental mathematics education [1, 5].

The magnitude of the observed pre–post difference indicates that the score increase was not only statistically significant but also substantial. In educational research, effect sizes provide information beyond statistical significance; however, their interpretation should consider the study design, outcome measure, and practical context rather than relying solely on conventional benchmarks [12, 16]. Accordingly, the large standardized pre–post difference observed in this study indicates a substantial increase in test performance among program participants. Nevertheless, this estimate should be interpreted in light of the single-group design and the study-specific assessment used in the present study.

### Effects across different proficiency levels

Improvements were observed across all three initial proficiency groups, and no statistically significant differences in gain scores were identified among the groups. Remedial education is often designed primarily for students with insufficient foundational knowledge; however, previous studies have suggested that revisiting fundamental concepts may also support learning among higher-achieving students [7].

The results of this study indicate that improved test performance was observed among participants in all three initial proficiency groups examined in the present sample. Previous studies have suggested that the effectiveness of educational interventions may vary depending on learners’ prior academic proficiency [24]. More recent evidence also indicates that the effects of remedial mathematics education may differ according to students’ initial mathematical preparation and the level of remediation provided [3].

### Unit-level learning characteristics

The unit-level analysis suggested that the magnitude of improvement differed across mathematical units, with larger gains tending to occur in areas where pre-test performance was lower.

In mathematics education, conceptual and procedural knowledge are closely related and may develop through reciprocal and iterative processes [21, 22]. The variation in improvement across units observed in this study may therefore partly reflect differences in the balance of procedural demands, conceptual understanding, and contextual interpretation required by the individual test items. The relatively low post-test performance on Q3 and Q4 may indicate that students continued to experience difficulty when multiple logarithmic properties had to be coordinated within a single calculation. However, because these items required relatively complex combinations of procedures, the results should not be interpreted as reflecting a general lack of understanding of logarithms. In contrast, relatively small improvements in some units may reflect a ceiling effect, as correct response rates were already relatively high in the pre-test.

### Implications for health science education

In health science education, mathematical ability and numeracy are considered essential for understanding specialized subjects and performing clinically relevant quantitative tasks [15, 17, 19]. Because concentration calculations and unit conversions are frequently required in clinical contexts, insufficient mathematical proficiency may affect students’ performance in such calculations and their engagement with specialized coursework.

Although the present program primarily focused on foundational mathematical skills, future iterations may benefit from incorporating more explicit links to medical technology and clinical engineering contexts. Contextualized basic skills instruction has been proposed as an approach for connecting foundational skills with disciplinary or occupational applications, thereby supporting the transfer of learning and student motivation [18]. More recent observational research has also reported positive associations between exposure to contextualized mathematics instruction and several postsecondary educational outcomes [28]. Such contextualization may help students understand the relevance of mathematical concepts to their subsequent coursework and professional practice. More broadly, providing learning support at the foundational stage of higher education may help establish a basis for subsequent specialized learning, while quantitative assessment of students’ performance may inform instructional design and the development of effective support strategies.

In recent years, the use of learning data for educational improvement has attracted increasing attention within the field of Learning Analytics [25]. The analysis of assessment data conducted in this study may be considered one example of such data-informed educational practice. These findings may be relevant to other health science and medical education programs in which students enter with diverse mathematical backgrounds and are expected to engage with quantitatively demanding coursework early in the curriculum.

### Limitations

This study has some limitations. First, the sample size was relatively small and limited to a single educational institution; therefore, caution is required when generalizing the findings. Observed learning outcomes may vary depending on student characteristics and institutional context. In addition, the criteria used to recommend participation differed between the two academic years because of the limited program capacity. This difference may have influenced the composition of the participant groups and should be considered when interpreting the pooled results. Moreover, a small number of students participated voluntarily, which may have introduced self-selection differences between these students and those for whom participation was strongly recommended.

Second, this study evaluated learning outcomes primarily using short-term test scores and did not examine longer-term learning retention or relationships with performance in specialized subjects. Moreover, because the study used a single-group pre-test/post-test design without a control group, causal interpretations should be made with caution, as observed changes may be influenced by alternative explanations other than the intervention itself [23].

Third, although this study analyzed performance at the unit level, learning in practice often involves relationships among multiple concepts. The results of the principal component analysis also suggest that learners’ understanding structures may not be fully represented by a simple one-dimensional scale.

The use of tests with identical structures may also have introduced practice effects.

### Future work

Future research should include validation across a broader range of educational institutions and student populations. In addition, combining assessment results with learning log data may enable more detailed analyses of learning processes.

Furthermore, analytical approaches that consider relationships among content units may provide deeper insights into learners’ cognitive structures. Multivariate analysis of educational data has the potential to generate useful information for improving instructional design and learning support strategies.

## Conclusions

In this study, we conducted statistical analyses using pre-test and post-test results to examine changes associated with remedial mathematics instruction implemented in the Department of Medical Technology and Clinical Engineering. The results showed statistically significant overall improvement in scores, accompanied by a large effect size, suggesting that the educational intervention was associated with improved foundational mathematics test performance.

Stratified analysis by proficiency level revealed significant improvements across all ability groups (Low, Middle, and High), indicating that pre–post improvements were observed in all three initial proficiency groups. These findings suggest that systematic review of foundational mathematical concepts may be associated with improved test performance across the three initial proficiency groups examined in the present sample. Furthermore, unit-level analysis showed improvements in correct response rates across multiple foundational content areas.

Relatively large improvements were observed in units where pre-test performance was low, suggesting that the program was associated with greater improvement in areas in which learners initially had difficulty. In health science education, foundational mathematical skills and numeracy are considered important for understanding specialized subjects. The findings of this study suggest that providing learning support during the early stages of higher education may help establish a foundation for subsequent specialized learning.

Although this study involved a relatively small sample from a single institution, and caution is required when generalizing the findings, the study provides a quantitative description of pre–post changes and unit-level performance patterns among program participants. Future research should include validation with more diverse student populations and examination of relationships between foundational academic skills and longer-term learning outcomes. Such investigations may contribute to a better understanding of the role of foundational education in health science curricula.

## Figures and Tables

**Fig. 4:**
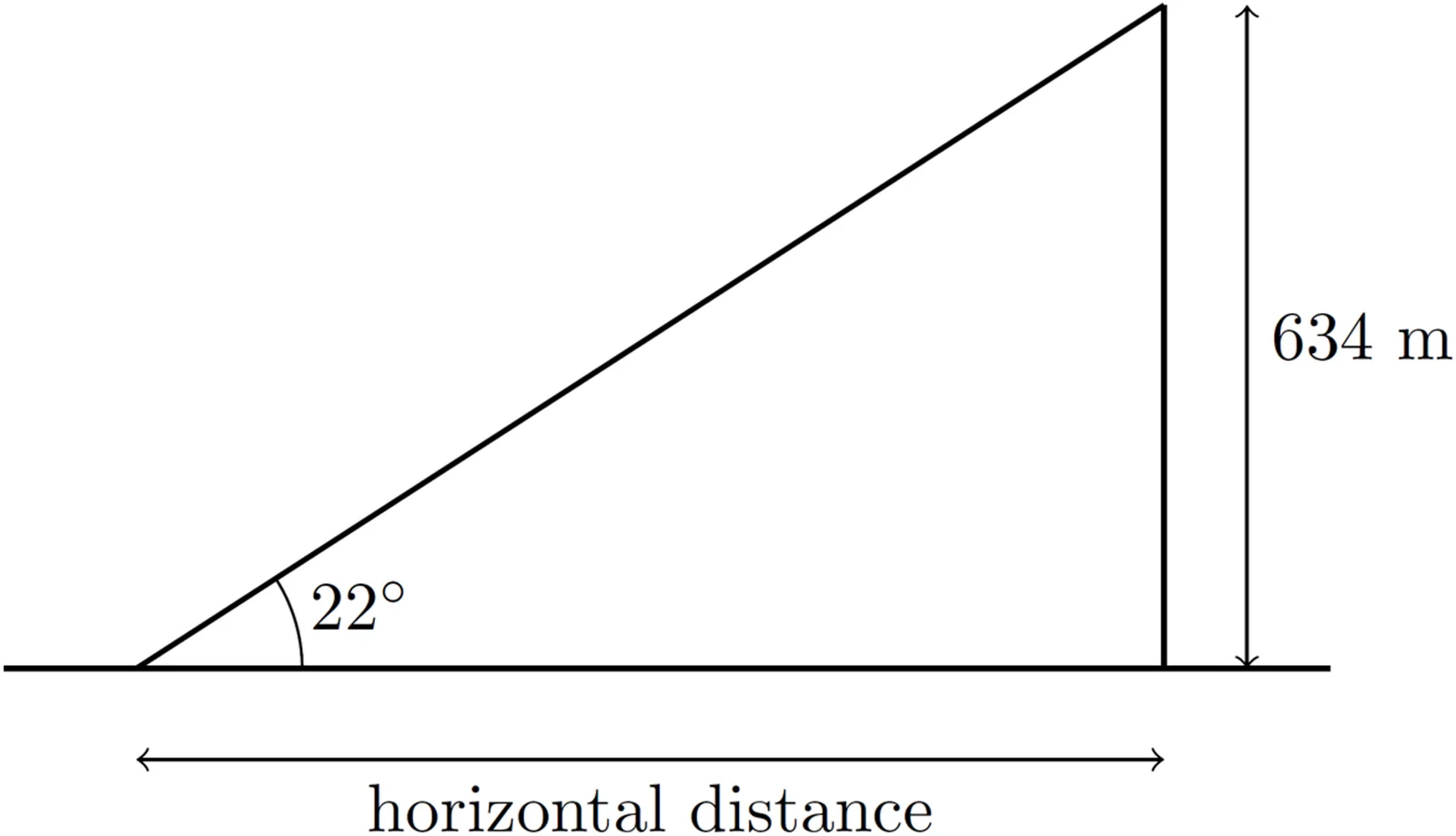
Schematic representation of the trigonometric-ratio problem

## Data Availability

The datasets generated and analyzed during the current study are not publicly available because they contain educational data that could potentially identify participants, but anonymized data may be available from the corresponding author on reasonable request and with permission from the institution.

## Appendix A: Representative Assessment Questions

The following items are representative questions from the pre-test. The post-test used different questions with the same structure and content domains.

### Concentration calculations

The following questions assessed students’ application of mathematical knowledge to chemistry-related contexts. Such knowledge is relevant to solution preparation related to life-support equipment in the Clinical Engineering program and to reagent preparation in the Medical Technology program.

#### Question 10. Answer the following questions.

1. How many grams of salt are contained in 300 g of a 12% salt solution?
2. A 200 g sample of a 15% salt solution, a 200 g sample of an 18% salt solution, and a 100 g sample of a 20% salt solution are mixed. What is the concentration of the resulting salt solution?
3. How many grams of water must be evaporated from 200 g of a 15% salt solution to obtain a 24% salt solution?

### Application of trigonometric ratios

The following question assessed students’ application of trigonometric ratios to physics- and engineering-related contexts. Trigonometric ratios are relevant to determining distances and angles in subjects related to medical technology and clinical engineering.

#### Question 11. Answer the following question.

A tower is 634 m high. From a point on level ground, the angle of elevation to the top of the tower is $22^\circ$. Approximately how far is the observation point from the base of the tower? Assume that $\tan 22^\circ = 0.40$. A schematic representation of the problem is shown in Fig. 4.

## Appendix B: Additional Results

Table 2 summarizes the complete unit-level results for the pooled sample. Pre and Post denote mean correct response rates, and Gain is expressed in percentage points. Raw *p*-values were obtained from paired *t*-tests, whereas adjusted *p*-values were calculated using the Holm method across the 11 unit-level comparisons. Statistical significance was determined using the Holm-adjusted *p*-values.

**Table 2:** Unit-level pre–post comparisons for the pooled sample

Unit	Pre (%)	Post (%)	Gain	*t*	Raw *p*	Adjusted *p*	*d*
Q1	53.9	62.3	8.4	1.92	0.060	0.060	0.25
Q2	44.0	65.1	21.1	4.37	*<0.001*	*<0.001*	0.57
Q3	4.3	44.8	40.5	7.62	*<0.001*	*<0.001*	1.00
Q4	22.0	46.6	24.6	5.32	*<0.001*	*<0.001*	0.70
Q5	18.1	68.1	50.0	9.93	*<0.001*	*<0.001*	1.30
Q6	35.3	57.8	22.4	5.02	*<0.001*	*<0.001*	0.66
Q7	11.2	59.9	48.7	8.33	*<0.001*	*<0.001*	1.09
Q8	14.1	68.7	54.6	11.92	*<0.001*	*<0.001*	1.57
Q9	5.2	51.7	46.6	6.60	*<0.001*	*<0.001*	0.87
Q10	41.1	73.0	31.9	6.98	*<0.001*	*<0.001*	0.92
Q11	15.5	59.5	44.0	5.58	*<0.001*	*<0.001*	0.73
